# Mechanistic Study of Release Characteristics of Two Active Ingredients in Transdermal Patch Containing Lidocaine−Flurbiprofen Ionic Liquid

**DOI:** 10.3390/pharmaceutics14102158

**Published:** 2022-10-10

**Authors:** Degong Yang, Xuejun Chen, Ziqing Li, Chunrong Yang

**Affiliations:** 1 Department of Pharmacy, Shantou University Medical College, No. 22 Xinling Road, Shantou 515041, China; 2 Guangdong Provincial Key Laboratory of Infectious Diseases and Molecular Immunopathology, Shantou University Medical College, No. 22 Xinling Road, Shantou 515041, China

**Keywords:** transdermal patch, ionic liquids, release characteristics, hydrogen bond, ionic hydrogen bond

## Abstract

Ionic liquids (ILs) have been proven to be an efficient technology for enhancing drug skin permeability. However, the question of whether the two components of ILs are released synchronously in transdermal preparations has remained unclear. Thus, this study aimed to investigate the release characteristics of two components of ILs and their underlying molecular mechanism. The ILs containing flurbiprofen (FLU) and lidocaine (LID) were synthesized and characterized. The four typical acrylates pressure sensitive adhesives (PSAs) with different functional groups were synthesized and characterized. The effects of PSAs on the release characteristics of two components of ILs were investigated by drug release tests and verified by skin permeation experiments. The action mechanisms were revealed by FTIR, Raman, dielectric spectrum, and molecular docking. The results showed that the average release amount of FLU (0.29 μmol/cm^2^) and LID (0.11 μmol/cm^2^) of ILs in the four PSAs was significantly different (*p* < 0.05), which illustrated that the two components did not release synchronously. The PSA−none and PSA−OH with low permittivity (7.37, 9.82) interacted with drugs mainly by dipole-dipole interactions and hydrogen bonds. The PSA−COOH and PSA−CONH_2_ with high permittivity (11.19, 15.32) interacted with drugs mainly by ionic bonds and ionic hydrogen bonds. Thus, this study provides scientific guidance for the application of ILs in transdermal preparations.

## 1. Introduction

The transdermal drug delivery system (TDDS) is becoming an ideal delivery system to replace conventional drug delivery routes, which delivers drugs through the skin into the systemic circulation to exert therapeutic effects [1]. However, due to the barrier function of stratum corneum (SC), the skin permeation amount of drug is difficult to meet the needs of treatment, and TDDS is still limited to drugs with relatively small molecular weight and moderate lipophilicity, which seriously restricts the rapid development of TDDS [2]. Recently, drug-ionic liquids (drug−ILs) have been proved to be a safe and efficient technology for enhancing drug skin permeability and have great potential and broad application prospects in the field of TDDS [3]. Previous studies mainly focused on the change of drug skin permeability in the solution system [4]. However, the transdermal preparation includes two processes: drug release and skin permeation [5]. Therefore, an investigation on the release characteristics of two active ingredients of drug−ILs is very important for scientific principles of drug−IL application in transdermal preparations.

ILs are organic salts formed by combining two ions with opposite charges under coulomb force, which are liquid below 100 °C [6]. Therefore, ILs can be designed at the molecular level by changing the types of anions or cations to achieve the desired purpose, including enhancing drug skin permeability [7]. For example, the skin permeation of etodolac was enhanced (9.3-fold) when etodolac was transformed into an IL using lidocaine [8]. ILs with α-linolenic and docosahexaenoic acid were more permeable through artificial skin membrane than the free base of donepezil, and the permeability coefficients were increased by 1.9- and 1.55-fold, respectively [9]. The previous studies proved that the change of drug skin permeability was mainly attributed to change of physicochemical parameters when drug-ILs were formed [10]. However, whether ILs remined stable, when they were added in transdermal preparation. The question of whether the two components are released synchronously remains to be clarified. It was also revealed that the fundamental mechanism of ILs was the presence of strong charge-assisted hydrogen bonds, van der Waals, and ionic interactions [11]. So, the addition of polymer is likely to break inter-ionic interaction of ILs and affect drugs release characteristics, thereby influencing skin permeation behavior. 

**Figure 1:**
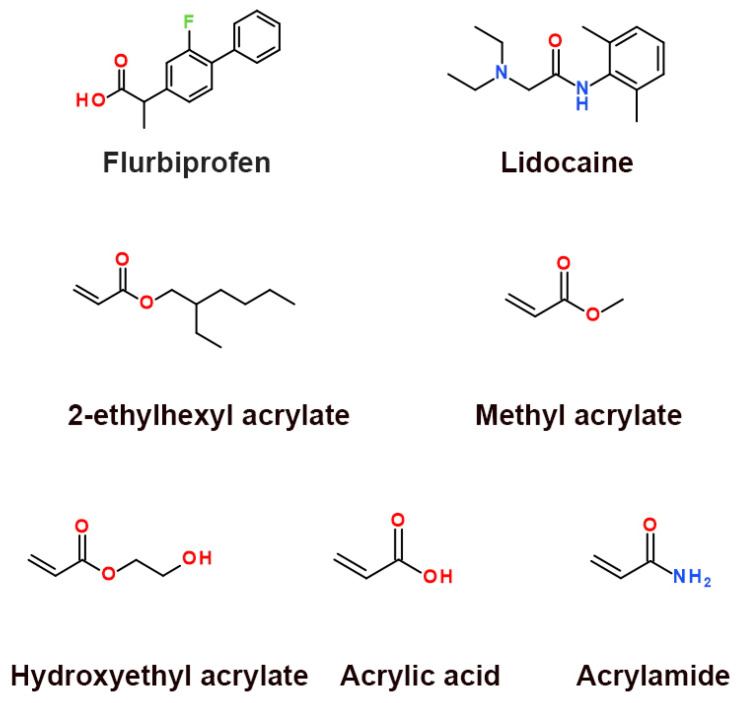
Chemical structures of model drugs and monomers (the chemical structures were obtained from the PubChem database).

In this study, the ILs were synthesized using flurbiprofen (FLU) and lidocaine (LID), because the clinical therapeutic effects of LID in combination with FLU for reducing pain were improved [12]. Their chemical structures are shown in Figure 1. In addition, the ILs were characterized by differential scanning calorimetry (DSC), Fourier transform infrared spectroscopy (FTIR), and nuclear magnetic resonance spectrometer (^1^H NMR). The four typical acrylates pressure sensitive adhesives (PSAs) with different functional groups were synthesized as model polymers, which almost covered commercial acrylate products. The monomers’ structures are shown in Figure 1. In addition, the PSAs were characterized by gel permeation chromatography (GPC), DSC, and FTIR. The effects of PSAs on the release characteristics of two active ingredients of ILs were investigated by in vitro drug release and verified by in vitro skin permeation experiments. The action mechanisms were revealed by FTIR, Raman, dielectric spectrum, and molecular docking. The study aimed to explore the release characteristics of two active ingredients in transdermal patches containing ILs and illustrate the underlying molecular mechanism, which can accelerate the development of transdermal preparations utilizing IL strategies.

## 2. Materials and Methods

### 2.1. Materials and Animals

FLU, LID, and caprylic/capric triglyceride (ODO) were purchased from Shanghai Macklin Biochemical Co., Ltd. (Shanghai, China). 2-ethylhexyl acrylate (EHA), methyl acrylate (MA), acrylic acid (AA), hydroxyethyl acrylate (HEA), acrylamide (ACR), and 2,2-Azobis were purchased from Aladdin Industrial Corporation (Shanghai, China).

Wistar rats (Male, 180–220 g) were provided by the laboratory animal center of Shantou University Medical College (Shantou, China). All operational processes were carried out according to the NIH Guidelines for the Care and Use of Laboratory Animals, and were approved by the Animal Ethics Committee of Shantou University Medical College.

### 2.2. Synthesis and Characterization of ILs

The ILs were synthesized using a salt metathesis reaction as follows. The FLU was added to LID-containing acetone (mole ratio of FLU/LID = 1:1) at room temperature for 2 h. The acetone was removed using a rotary evaporator, then the obtained ILs were placed in a vacuum drying chamber for 48 h. The glass transition temperature (Tg) of ILs was investigated by the Mettler-Toledo thermal analyzer (DSC, Mettler-Toledo International Inc., Zurich, Switzerland) from −60 °C to 60 °C with a heating rate of 5 °C/min. The structure of the ILs was investigated by ^1^H NMR spectroscopies (Bruker AVANCE III 600 MHz, Karlsruhe, Germany) and FTIR (Bruker Vertex 70 spectrometer, Billerica, MA, USA) in the range 400–4000 cm^−1^ with 128 scans.

### 2.3. Synthesis and Characterization of PSAs

Four PSAs were synthesized by radical polymerization reactions. Firstly, the various monomers were blended under stirring at 80 °C in a N_2_ atmosphere (PSA−none, 2-EHA:MA = 65:35; PSA−OH, 2-EHA:MA:HEA = 65:30:5; PSA−COOH, 2-EHA:MA:AA = 65:30:5; PSA−CONH_2_, 2-EHA:MA:ACR = 65:30:5). Then, 2,2-Azobis was added and reacted for 12 h. The molecular weight of PSAs was investigated by gel permeation chromatography (Isocratic HPLC Pump1515, Refractive Index detector 2414, Waters Co., Milford, MA, USA). The Tg of PSAs was investigated by the Mettler-Toledo thermal analyzer (DSC, Mettler-Toledo International Inc., Greifensee, Switzerland) from −69 °C to 0 °C with a heating rate of 5 °C/min. The structure of the PSAs was investigated by ^1^H NMR spectroscopies (Bruker AVANCE III 600 MHz, Germany).

### 2.4. The Skin Permeability of ILs in Solution System

To confirm the enhancement action of ILs, the skin permeability of two active ingredients of ILs was investigated by skin permeation experiment of drug solution system (*n* = 4). The process of skin was in accordance with the literature [13]. The FLU, LID, and ILs were dissolved in ODO (dielectric constant: 2.3) and used as donor fluid, and PBS (pH 7.4) was used as the receptor fluid. The medias were stirred at 600 rpm and maintained at 32 °C. Sampling time points were 2, 4, 6, 8, and 12 h. At each sampling point, 2.0 mL of receptor solution was withdrawn, and the same volume of PBS buffer was added to maintain constant volume. Drug concentration was determined with the Shimadzu UPLC system (pump LC-30AD, UV-VIS detector SPD-20 A/20 AV) and a Shim-pack GIST C18-AQ HP (1.9 μm, 100 × 2.1 mm). Mobile phase and wavelength of drugs were as follows: FLU (Methanol:water:acetic acid = 75:25:0.5, 245 nm) and LID (Acetonitrile:water:phosphoric acid:triethylamine = 20:80:0.1:0.1, 220 nm).

### 2.5. In Vitro Drug Release of Patch

The effects of PSAs on the release characteristics of two active ingredients of ILs were investigated by in vitro drug release experiments. The transdermal patches were prepared by the solvent evaporation method and summarized as follows. The FLU, LID, and ILs were dissolved in ethyl acetate, respectively. Then, the PSAs were added to obtain homogeneous solutions (drug loading: 3%). Afterwards, the solutions were coated onto release liner using a film applicator and dried (room temperature for 10 min and oven-dried at 50 °C for 10 min). Finally, the backing film was used to obtain patches. The in vitro drug release experiments were operated by a horizontal diffusion cell and semipermeable membrane (Cellophane^®^) at 32 °C (*n* = 4). The PBS (pH 7.4) was used as accept fluid. Sampling time points were 1, 2, 4, 6, 8, and 12 h. The quantitative methods of drugs were the same as in Section 2.4. 

### 2.6. In Vitro Skin Permeation of Patch

The drug release results were verified by skin permeation experiments (*n* = 4). Rat skin was used, replacing the semipermeable membrane, and other experimental processes were in accordance with the drug release experiment.

### 2.7. Mechanisms of Action

#### 2.7.1. FTIR

The FTIR spectroscopy was used to explore the interaction between ILs and PSAs. The blank PSAs, drug−PSAs, and ILs−PSAs were weighed and dissolved in ethyl acetate, then added to KBr pellet. The scan range was set from 400 to 4000 cm^−1^ with a resolution of 4 cm^−1^, and 128 scans were performed for each measurement.

#### 2.7.2. Raman

The Raman spectra were collected to study the molecular details between ILs and PSAs using a Renishaw inVia Laser Micro Raman spectrometer, equipped with an excitation laser operating at 785 nm with a laser power setting of 300 mW. The blank PSAs, drug-PSAs, and ILs-PSAs were placed in front of the 50 × objective lens. Data acquisition time was 10 s and were processed by PeakFit 4.0 software.

#### 2.7.3. Dielectric Spectrum

The dielectric permittivity (ε) of PSAs was measured to evaluate the polarity using the broadband dielectric spectrometer (Novocontrol Technologies, Montabaur, Germany) [14]. Dielectric measurements were conducted at 32 °C in the frequency range of 10^−1^–10^7^ Hz. The PSA samples (thickness: 1 mm) were placed between two round copper electrodes (20 mm diameter) and a PTFE spacer (thickness, 1 mm; area, 59.69 mm^2^; capacity, 1.036 pF). The dielectric permittivity consisted of real (ε′) and imaginary (ε″) components, which was equal to the low frequency limit (ω → 0) of ε′(ω).

#### 2.7.4. Molecular Docking

Molecular docking was used to provide precisely useful information about the molecular interaction between ILs and PSAs by the software package Materials Studio (version 7.0, Accelrys Inc., San Diego, CA, USA). Structures of drugs and monomers were obtained from the PubChem database and structures of ILs and PSAs were built according to synthesized ILs and PSAs. Then, geometry optimization was performed, employing the Forcite module and COMPASSII force field [15]. After that, molecular docking was performed in the Blends module based on the modified Flory–Huggins theory and the best docking type was obtained according to binding energy scores [16].

### 2.8. Statistical Analysis

Results were expressed as mean ± S.D. ANOVA was used to determine whether there was a significant difference between the release amounts of FLU and LID of the ILs in the PSAs by SPSS 21.0 software. A significant level was taken as *p* < 0.05. All data were checked for normality and homoscedasticity before the ANOVA.

## 3. Results

### 3.1. Characterizations of ILs 

**Figure 2:**
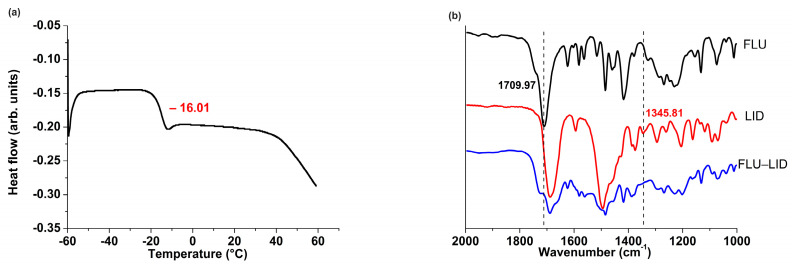
(**a**) Glass transition temperatures (*T*_g_) of IL; (**b**) FTIR spectra of FLU, LID, and FLU−LID.

The synthesized ILs were clear and viscous liquids at room temperature (*T*_g_: −16.01 °C), whereas FLU and LID were white powders. Characteristic peaks of C=O stretching vibration peak of FLU (1709.97 cm^−1^) and C−N stretching vibration peak of LID (1345.81 cm^−1^) were observed in the FTIR spectra (Figure 2b). Their peak intensity significantly decreased when ILs were formed, which illustrated that the COOH of FLU was deprotonated into COO^−^ [8]. ^1^H NMR was also used to assess the degree of proton transfer and the results was consistent with that of FTIR. As shown in Table 1, the ^1^H chemical shifts for anion (FLU) shifted upfield, and the opposite trend was observed for the cations (LID). The COOH of the FLU was a strong electron withdrawing group, which had an electron withdrawing inductive effect on the connected CH_2_ and CH_3_, thereby causing a decrease in the density of the electron cloud and shift downfield [17]. When ILs were formed, proton transfer occurred between the COOH of FLU and N of LID, thereby weakening the deshielding effect and making the CH_2_ and CH_3_ shift upfield.

### 3.2. Characterizations of PSAs

**Figure 3:**
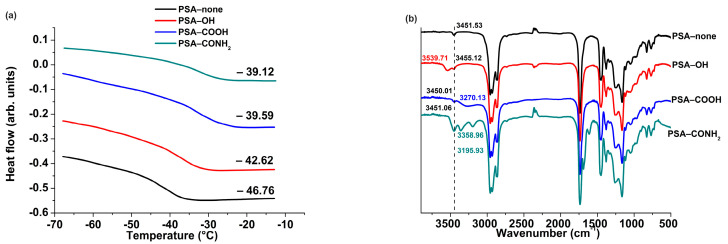
(**a**) Glass transition temperatures (*T*_g_) of four PSAs; (**b**) FTIR spectra of the characteristic peaks of four PSAs.

Four representative PSAs were designed and synthesized in order to investigate their effects on the release characteristics of two active ingredients of ILs. As shown in Table 2, number average molecular weight (*M*_n_), weight average molecular weight (*M*_w_), and polydispersity index (*PDI*) were measured. The *T*_g_ values of PSAs were measured by DSC (Figure 3a), and the rank order was follows: PSA−none (−46.76) < PSA−OH (−42.62) < PSA−COOH (−39.59) < PSA−CONH_2_ (−39.12). It indicated that PSA−COOH and PSA−CONH_2_ had poor mobility of polymer chain, which is mainly attributed to the strong intermolecular interaction between the main chains of PSAs [13,18]. FTIR is a powerful tool and was used to characterize the structures of PSAs. As shown in Figure 3b, the characteristic peak at 3449.53 cm^−1^ was attributed to C=O overtone peak of PSA−none. The characteristic peaks at 3455.12 and 3539.71 cm^−1^ were attributed to OH stretching vibration and C=O overtone peaks of PSA−OH, respectively. The characteristic peaks at 3270.13 and 3450.01 cm^−1^ were attributed to O−H stretching vibration and C=O overtone peaks of PSA−COOH, respectively. The characteristic peaks at 3195.93, 3358.96, and 3451.06 cm^−1^ were attributed to amide N−H symmetric and antisymmetric stretching and C=O overtone peaks of PSA−CONH_2_, respectively.

### 3.3. The Skin Permeability of ILs

**Figure 4:**
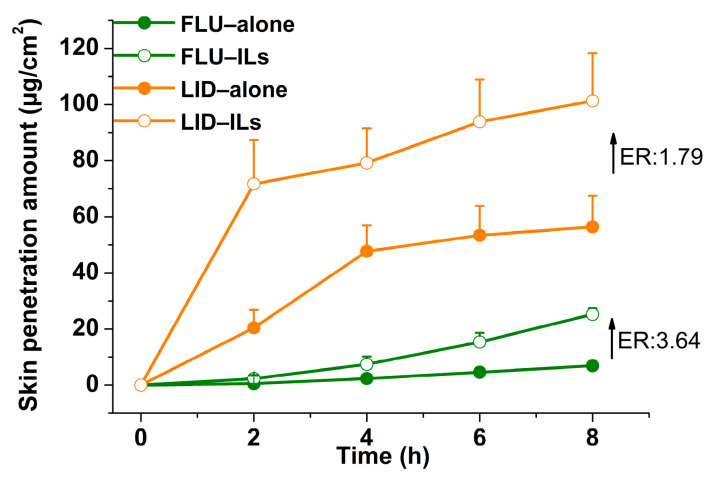
The skin permeability of FLU, LID and ILs (*n* = 4).

In order to compare the skin permeability of FLU and LID of ILs with that of FLU and LID alone, in vitro skin permeation tests were carried out. As seen in Figure 4, the skin penetration amounts of drug−ILs were greater than that of drugs alone, and the enhancement ratios (*ER* = *Q*_durg-ILs_/*Q*_drug-alone_) were 3.64 and 1.79 for FLU and LID, respectively. The results proved the advantages of IL technology in improving the transdermal permeability of drugs, which provides the precondition for investigating whether drug−ILs played an equal role when they are applied in transdermal patches.

### 3.4. In Vitro Drug Release Results of Patch

**Figure 5:**
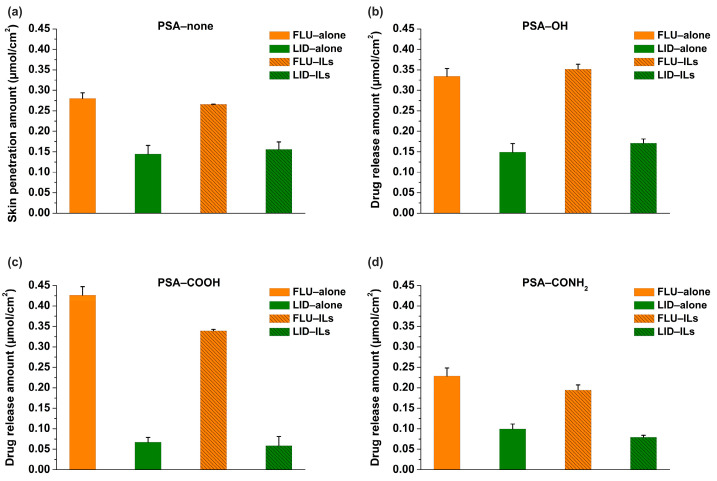
The in vitro release results of FLU, LID and ILs in the PSA−none (**a**), PSA−OH (**b**), PSA−COOH (**c**), and PSA−CONH_2_ (**d**) (*n* = 4).

Drug release experiments were performed to investigate the impacts of PSA on the release characteristics of two active ingredients of ILs. To clearly illustrate whether the two components are released synchronously, the units of drug release amount were set as μmol/cm^2^ because of the mole ratio of synthetic ILs (1:1). As shown in Figure 5, two main phenomena were found. Firstly, the release amounts (*R*) of FLU and LID of ILs in the four PSAs were significantly different (*p* < 0.05), which indicated that the two drugs of ILs are released independently. Additionally, the *R*_FLU_ of ILs was all higher than the *R*_LID_ of ILs in the PSAs; this is because the LID was a basic drug and had a higher polarizability (28.4) [13,18]. The specific results are as follows: for the PSA−none, the *R*_FLU_ and *R*_LID_ of ILs were 0.27 and 0.15, respectively; for the PSA−OH, the *R*_FLU_ and *R*_LID_ of ILs were 0.35 and 0.17, respectively; for the PSA−COOH, the *R*_FLU_ and *R*_LID_ of ILs were 0.34 and 0.06, respectively; for the PSA−CONH_2_, the *R*_FLU_ and *R*_LID_ of ILs were 0.19 and 0.08, respectively. Secondly, in order to investigate the effect of IL formation on drug release, the *D*_drug-alone_ and *D*_drug-ILs_ were compared. The results showed that drug release amounts of FLU and LID in the PSA−none and PSA−OH did not change remarkably when ILs were formed. However, drug release amounts of FLU in the PSA−COOH (*D*_FLU-alone_: 0.43; *D*_FLU-ILs_: 0.33) and PSA−CONH_2_ (*D*_FLU-alone_: 0.23; *D*_FLU-ILs_: 0.18) both decreased, and drug release amounts of LID in the PSA−COOH (*D*_LID-alone_: 0.06; *D*_LID-ILs_: 0.05) and PSA−CONH_2_ (*D*_LID-alone_: 0.09; *D*_LID-ILs_: 0.08) remained stable. This was possibly because the PSA−COOH and PSA−CONH_2_ were both deprotonated [19].

### 3.5. In Vitro Skin Penetration Results of Patch

**Figure 6:**
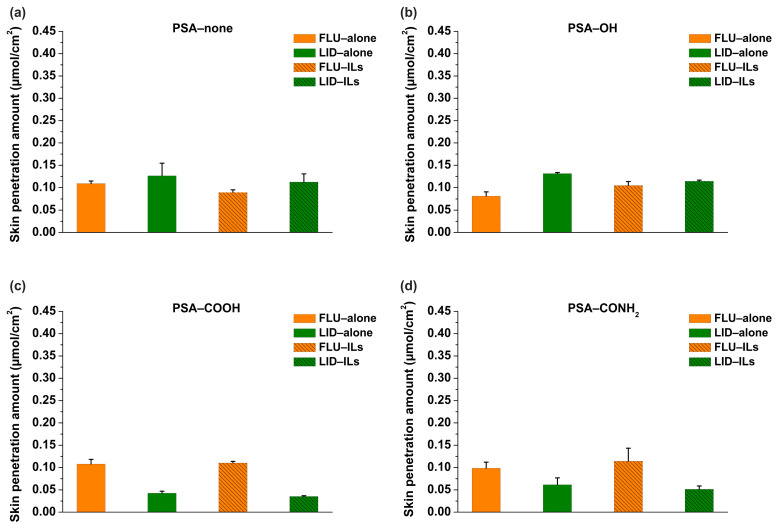
The in vitro skin permeation results of FLU, LID and ILs in the PSA−none (**a**), PSA−OH (**b**), PSA−COOH (**c**), and PSA−CONH_2_ (**d**) (*n* = 4).

To identify the in vitro drug release trends, in vitro permeation studies using rat skin were conducted. The rate limiting step of TDDS was described as follows: *F*_S/R_ = S/R (S: skin penetration amount) [20]. So, the calculated *F*_S/R_ values of FLU and LID were about 0.40 and 0.88, respectively. It illustrated that the drug delivery of LID was entirely controlled by drug release process, but the skin made contributions to control process for the FLU. Based on that, the skin penetration results of drugs were summarized (Figure 6). Firstly, the *S* vales of FLU and LID of ILs in the PSA−none and PSA−OH were similar. It was principally because the skin permeability of FLU was poor, which caused its skin penetration amounts to all decrease. However, the *S* values of FLU and LID of ILs in the PSA−COOH and PSA−CONH_2_ were significantly different, which were in agreement with drug release amounts. It was principally because the LID hardly released from PSA−COOH and PSA−CONH_2_, owing to ionic bonds [21]. Secondly, the *S*_drug-alone_ and *S*_drug-ILs_ were also compared. The results showed that *S* vales of FLU and LID in the four PSAs did change remarkably when ILs were formed, which were almost in agreement with the drug release results, expect for *S*_FLU-alone_ and *S*_FLU-ILs_ in PSA−COOH and PSA−CONH_2_, owing to the poor permeability of FLU.

### 3.6. Mechanisms of Action

#### 3.6.1. FTIR

**Figure 7:**
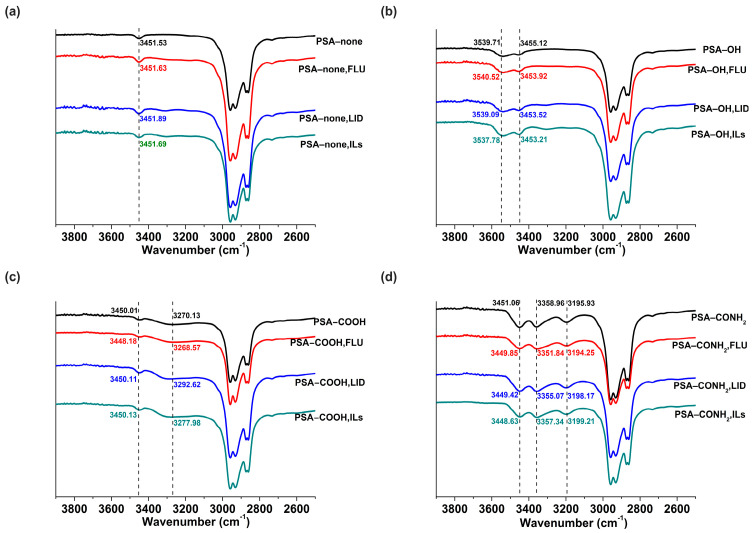
FTIR spectra in C=O overtone peak for the PSA−none (**a**), C=O overtone peak and OH stretching vibration peak for the PSA−OH (**b**), C=O overtone peak and O−H stretching vibrational peak of COOH for the PSA−COOH (**c**), C=O overtone peak, antisymmetric and symmetric stretching of the N−H for the PSA−CONH_2_ (**d**).

In order to determine the interactions between drugs and PSAs, FTIR spectra of the blends and their individual ingredients were analyzed. For the PSA−none, the additions of FLU, LID, and ILs made the C=O overtone peaks of PSA−none at 3451.53 cm^−1^ move to 3451.63, 3451.89, and 3451.69 cm^−1^, respectively, which indicated that the dipole-dipole interaction played a major role between drugs or ILs and C=O of PSA−none (Figure 7a) [22]. For the PSA−OH, significant changes were observed in the C=O overtone band located at 3455.12 cm^−1^. The additions of FLU, LID, and ILs made the peak shift to 3453.92, 3453.52, and 3453.32 cm^−1^, respectively (Figure 7b). The red shift indicated hydrogen bonds between drugs or ILs and the carbonyl group of PSA−OH [23]. For the PSA−COOH, significant changes were observed in the O−H stretching vibration band of COOH located at 3270.13 cm^−1^. The additions of FLU, LID, and ILs made the peak shift to 3268.57, 3292.62, and 3277.98 cm^−1^, respectively (Figure 7c). The red shift indicated hydrogen bonds between FLU and the O−H of PSA−COOH. The blue shift indicated that COOH in PSA was partially deprotonated into COO^−^, and deprotonated COO^−^ was involved in strong ionic hydrogen bonds with LID or ILs [11]. A similar shift was also found in the PSA−CONH_2_. The characteristic absorption band at 3195.93 cm^−1^ assigned to N−H symmetric stretching was chosen as the representative. The additions of FLU, LID, and ILs made the peak shift to 3194.25, 3198.17, and 3199.21 cm^−1^, respectively (Figure 7d). The results illustrated that hydrogen bond interactions between FLU and PSA−CONH_2_ existed, and weak ionic hydrogen bonds between LID or ILs and PSA−CONH_2_ were presented, compared with PSA−COOH according to characteristic peak displacement values.

#### 3.6.2. Raman 

**Figure 8:**
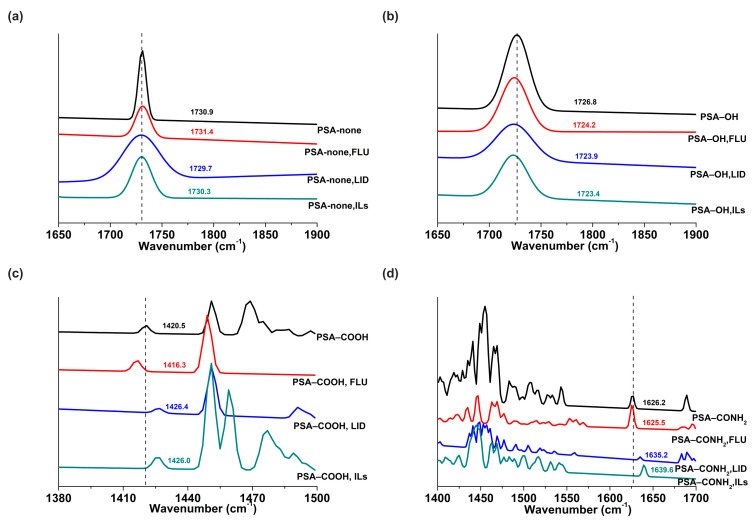
Raman spectra in C=O stretching vibration peak for the PSA−none (**a**) and PSA−OH (**b**), O−H out-of-plane bending vibration peak of COOH for the PSA−COOH (**c**), the N−H stretching vibration peak for the PSA−CONH_2_ (**d**).

Raman analysis was used to have a better insight into the molecular level of structural interaction between drugs and PSAs when ILs were formed. For the PSA−none, the additions of FLU, LID, and ILs made the C=O stretching vibration peak of PSA−none at 1730.9 shift to 1731.4, 1729.7, and 1730.3 cm^−1^, respectively, indicating the existence of dipole-dipole interactions between drugs or ILs and C=O of PSA−none (Figure 8a). For the PSA−OH, the band at 1726.8 cm^−1^ was red-shifted by 2.6, 2.9, and 3.4 cm^−1^ with the additions of FLU, LID, and ILs, which were attributed to the hydrogen bond interactions (Figure 8b). For the PSA−COOH, the O−H out-of-plane bending vibration peak of COOH was located at 1420.5 cm^−1^, and shifted to 1416.3, 1426.4, and 1426.0 cm^−1^ with the additions of FLU, LID, and ILs (Figure 8c) [24]. It indicated that PSA−COOH interacted with FLU by hydrogen bonds and interacted with LID or ILs by ionic hydrogen bonds. A similar phenomenon was also observed in the PSA−CONH_2_. The N−H stretching vibration peaks at 1626.2 cm^−1^ shifted to 1625.6, 1635.2, and 1639.6 cm^−1^ with the additions of FLU, LID, and ILs (Figure 8d) [25]. Above all, the results of Raman were in good agreement with the FTIR results.

#### 3.6.3. Dielectric Spectrum

**Figure 9:**
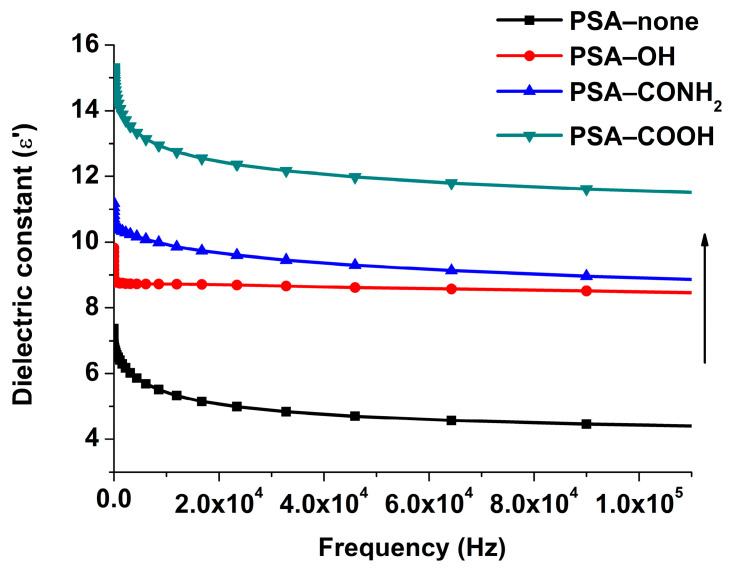
Normalized dielectric constant ε′ as a function of frequency of the PSA−none, PSA−OH, PSA−COOH, and PSA−CONH_2_.

The interaction nature between ILs and PSAs was dependent on the state of ILs in the PSAs, which was related to the polarity of PSAs [26]. The dielectric permittivity values were determined by measuring real permittivity as a function of frequency and the results are shown in Figure 9. The dielectric permittivity was ranked as follows: PSA−none (7.37) < PSA−OH (9.82) < PSA−CONH_2_ (11.19) < PSA−COOH (15.32), which showed that PSA−CONH_2_ and PSA−COOH were at higher polarity, and the ILs tended to dissociate with increased polymer polarity. On the other hand, with increasing dielectric permittivity, polymer segmental dynamics arising from the intra- and interchain polar interactions were slowed [27].

#### 3.6.4. Molecular Docking

**Figure 10:**
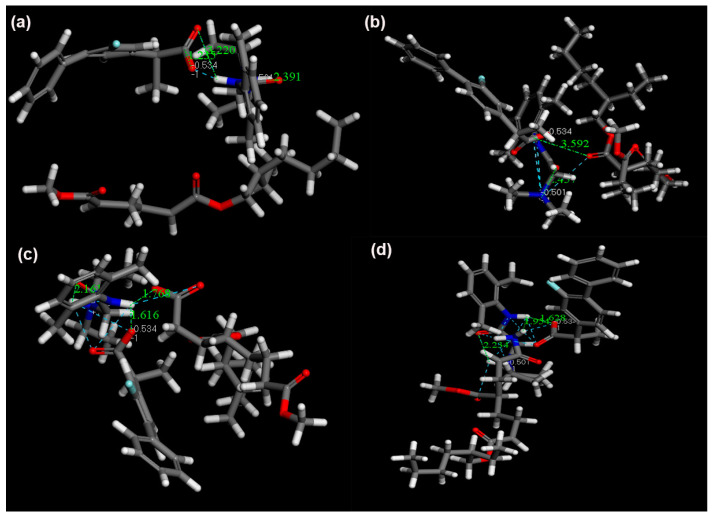
Optimal conformations of ILs with (**a**) PSA−none, (**b**) PSA−OH, (**c**) PSA−COOH, and (**d**) PSA−CONH_2_.

Molecular docking was used to investigate the interaction between ILs and PSAs. Figure 10a showed that hydrogen bonding was not present in the complexes of ILs and PSA−none. Figure 10b showed that hydrogen bonding was present in the complexes of ILs and PSA−OH (Distances: 3.592 Å). Figure 10c,d showed that the distances between the ILs and PSA−COOH and PSA−CONH_2_ were 1.616 and 1.954 Å, respectively. Above all, it was concluded that the order of interaction strength was: ILs−PSA−none < ILs−PSA−OH < ILs−PSA−CONH_2_ < ILs−PSA−COOH, which was in agreement with the results of FTIR and Raman [28].

## 4. Discussion

ILs show great potential for TDDS by enhancing the permeability of the drug, but the question about release characteristics of two active ingredients of ILs in transdermal preparations has not been settled [29]. To this end, this study systematically investigated the effects of four representative PSAs on the drug release of ILs, thereby illustrating whether the two components are released synchronously. Unexpectedly, the release amounts of FLU and LID of ILs in the four PSAs were all different, which illustrated that the two drugs released independently. Furthermore, the effects of IL formation on drug release from different PSAs were different, and were mainly divided into two categories according to polarity of PSAs (PSA−none and PSA−OH at the low polarity; PSA−COOH and PSA−CONH_2_ at the high polarity). Overall, this study provided a solid theoretical basis for the rational application of ILs in transdermal preparations.

The transdermal patch as representative transdermal preparation was chosen for the next study, which included two processes: drug release and skin permeation. The ILs containing FLU and LID were synthesized and characterized, and skin permeability experiments showed that the skin penetration amounts of the two drugs were enhanced by IL technology (Figure 4). The four PSAs containing different functional groups were chosen for investigating the effects of polymers on drug release because the interaction nature and strength between ILs and polymers is dependent on the types of the donor and acceptor formed [30].

For the PSA−none and PSA−OH, the *D* values of FLU and LID of ILs were significantly different, and *D*_drug-alone_ and *D*_drug-ILs_ did not make a remarkable change (Figure 5). The drug release results were also confirmed by skin penetration experiments (Figure 6) because the study showed a strong correlation between the in vitro % permeation across human skin and in vivo % absorption of estradiol [31]. Thus, in vivo tests were not performed in the study. The above results indicated that the PSA−none and PSA−OH did not break the inter-ionic interaction of ILs, which were further proved by FTIR, Raman, and molecular docking. The FTIR and Raman results showed that the C=O peaks of PSA-none did not shift with the addition of drugs or ILs. Additionally, there was no hydrogen bonding between ILs and PSA−none according to the results of molecular docking. It suggested the dominance of dipole-dipole interactions [32]. Furthermore, weak dipole-dipole interactions between ketoconazole and polyvinylpyrrolidone resulted in amorphous solid dispersion with higher crystallization propensity [33]. For the PSA−OH, the red shift of C=O band was observed in the FTIR and Raman results and the distance between C=O of PSA−OH and ILs was 3.592 Å (Figure 10), which was thought to be due to hydrogen bonds. It was reported that curcumin made OH stretching at 3456 cm^−1^ of hydroxypropyl methyl cellulose shift to 3445 cm^−1^, indicating the formation of the hydrogen bond between the polymer and curcumin [34]. The underlying molecular mechanism was revealed by dielectric spectrum. The dielectric permittivity of PSA−none and PSA−OH were 7.37 and 9.82, which indicated that they were both at low polarity. At low polarity, ion transport in the system was limited by ion dissociation into the polymer medium, and low dielectric polymer were expected to have ionic conductivity limited by IL dissociation [35].

For the PSA−COOH and PSA−CONH_2_, the *D* values of FLU and LID of ILs were significantly different. The *D*_LID-ILs_ was close to *D*_LID-alone_, but *D*_FLU-ILs_ was smaller than *D*_FLU-alone_ (Figure 5). The results implied that the PSA−COOH and PSA−CONH_2_ participated in inter-ionic interaction of ILs. For the LID (alkaline drug) with low *D* (<0.09), ionic bonds were formed between the PSAs and drugs whether the ILs were formed or not, thereby causing LID to be irreversibly bonded to the PSAs [21]. It was interesting that the release amounts of FLU decreased when ILs were formed. This is attributed to the presence of ionic hydrogen bonds and proved by FTIR and Raman results. The blue shifts of characteristic peaks in the PSA−COOH and PSA−CONH_2_ showed strong ionic hydrogen bonds between FLU−ILs and PSAs, which were opposite with the red shifts when the FLU was separately added into PSAs [36]. This is mainly owing to the deprotonation of PSA−COOH and PSA−CONH_2_, thereby it took part in inter-ionic interactions of ILs (Distance: 1.616 and 1.954 Å) (Figure 10) [16]. More importantly, the dielectric permittivity of PSA−COOH and PSA−CONH_2_ were 15.32 and 11.19, which were at higher polarity. On one hand, high dielectric permittivity was expected to have higher potential for ion solubility [27]. On the other hand, stronger polymer–ion interactions slowed polymer segmental dynamics with increases in polarity [37].

However, there were still some limitations in the study. Firstly, the four PSAs used in this study were relatively lipophilic, and ILs were relatively stable in the PSAs. However, whether the obtained conclusions are workable to hydrophilic polymer matrix remains to be investigated, because the previous study proved the instability of ion-pair complexes due to their dissociation in the hydrophilic viable epidermis [38]. As a result, the effects of hydrophilic polymer on release characteristics of two active ingredients of ILs will be studied in the future.

## 5. Conclusions

In this study, the release characteristics of two active ingredients of ILs in the PSAs with different function groups were elucidated systemically, and their underlying action mechanisms were revealed clearly from the perspective of intermolecular interactions. It was found that the release amounts of FLU and LID of ILs in four PSAs were all significantly different, which proved that the two components were not released synchronously. The drug release amount directly determined its skin penetration amount. The PSA−none and PSA−OH with low polarity interacted with drugs by dipole-dipole interactions and hydrogen bonds. The PSA−COOH and PSA−CONH_2_ with high polarity interacted with drugs by ionic bonds and ionic hydrogen bonds. Thus, this work provides a valuable basis for IL application in transdermal preparations and helps to accelerate the development of ILs in TDDS.

## Figures and Tables

**Table 1:** ^1^H NMR data of FLU, LID and FLU-LID ILs (Chemical shift, ppm).

Characteristic Peak	FLU	LID	FLU-LID
−COOH	11.24	—	Disappear
−COOH−CH_2_	3.77	—	3.70
−COOH−C−CH_3_	1.54	—	1.49
−N−CH_2_−C=O	—	3.21	3.42
−N−CH_2_	—	2.68	2.80
−N−C−CH_3_	—	1.11	1.16

**Table 2:** Physicochemical parameters of synthesized PSAs.

PSAs	*M*_W_ (Da)	*M*_n_ (Da)	*PDI* ^a^
PSA−none	521,503	161,069	3.23
PSA−OH	530,435	141,788	3.74
PSA−COOH	578,815	205,511	2.82
PSA−CONH_2_	680,512	215,870	3.15

^a^ Polymer dispersity index.
